# Sea urchin (*Diadema savignyi)* extract as a novel protective agent against cisplatin induced neurotoxicity in rats

**DOI:** 10.1186/s40360-023-00651-4

**Published:** 2023-02-23

**Authors:** Eman A. Khalil, Hamdy Swelim, Hala El-Tantawi, Ahmed Abdellatif

**Affiliations:** 1 Department of Biology, School of Sciences and Engineering, The American University in Cairo, Cairo, 11835 Egypt; 2 Department of Zoology, Faculty of Science, Ain Shams University, Cairo, Egypt

**Keywords:** Neurotoxicity, Cisplatin, Cisplatin induced toxicity, GFAP, Bcl2, Sea urchins, Oxidative stress, *Diadema Savignyi*

## Abstract

Neurotoxicity is a severe side effect of platinum compounds used for cancer chemotherapy such as Cisplatin. This neurotoxicity leads to severe cognitive and nervous dysfunction, therefore, limiting the dose of Cisplatin and compromising the treatment protocol.

The present study investigates the neuroprotective effect of Sea Urchins which is a marine animal known for its rich bioactive compounds. Male Sprague Dawley rats received Cisplatin (2 mg/kg body weight) for 4 weeks, two times per week, followed by Sea Urchin extracts (50 and 100 mg/kg body weight) twice weekly for 4 weeks.

Results show that rats treated with Urchin’s extracts showed a significant improvement in the thermal (heat and cold) sensitivity compared to untreated rats. Liver enzymes Alanine Aminotransferase (ALT) and Aspartate Aminotransferase (AST) and Urea levels were also significantly decreased back to normal following treatment with sea urchin extracts. Brain tissue oxidative stress marker Nitric oxide (NO) and lipid peroxidation marker Malondialdehyde (MDA) increased significantly in the cisplatin-treated rats while the reduced glutathione levels (GSH) and catalase activity (CAT) showed a significant decrease. Treatment with sea Urchin extracts reversed these changes.

Histological and immunohistochemical examination of the cerebral cortex reveled degenerative changes such as karyopyknosis and shrunken necrotic ghost like neurons in the cisplatin treated groups. There was also strong positive Glial fibrillary acidic protein (GFAP) reactivity and a negative B-cell leukemia/lymphoma 2 protein (Bcl2) reaction in most apparent neurons, indicating strong apoptotic changes. Treatment with Urchin extracts reversed these changes. Quantification of cerebral cortex neurons also revealed the strong effect of the extracts. Cisplatin treated groups showed 3708 cells/ mm3 compared to 8091 cells/mm^3^ in the normal rats. Extract treatment increased the neuronal numbers to almost normal levels. Quantification of the Immuno-histochemical expression of GFAP showed an increase by 10-folds after cisplatin administration. A remarkable decline from the cisplatin group was seen in the extract treated groups.

In Conclusion, Sea Urchins extracts possess a strong neuroprotective activity and could provide a novel therapeutic method to prevent Cisplatin-induced neurotoxicity.

## Introduction

Cisplatin is a heavy metal commonly used in cancer chemotherapy to treat lung, ovary, and testicular cancers [1]. Accumulation of Cisplatin causes toxicity in various body organs, such as neurotoxicity, cardiotoxicity, nephrotoxicity, and ototoxicity. Such toxicity limits the dose of cisplatin and reduces its chemotherapeutic efficacy [2, 3]. Other neurotoxins include commonly used chemicals such as Organophosphates, Carbamates, Pyrethroids, Organochlorines, insecticides, pesticides, and heavy metals such as mercury and lead [4, 5]. Neurotoxicity effects depend on different factors such as the characteristics of the neurotoxin, its dose, exposure time, and the ability of the nervous tissue to metabolize and excrete the toxin [4].

Cisplatin-induced neurotoxic effects cause impairments in cognition and executive function [6–8]. Two primary mechanisms are implicated in neurotoxicity, mitochondrial dysfunction and oxidative stress [9]. DNA damage and apoptosis were also reported with cisplatin and confirmed by electrophysiological and histopathological experiments [1, 10–12]. The outcome of neurotoxicity depends on the duration and extent of exposure to the toxic substance and the degree of neural damage [13].

Natural products provide a promising source of therapeutics due to their high chemical diversity, which makes them favorable for future drug development [14, 15]. Most of the drugs on the market come from natural sources. Marine animals are rich in bioactive compounds. Several marine-derived compounds have been isolated and characterized with promising biological activities. Many marine extracts’ therapeutic and pharmacological profiles suggest their potential as antitumor, anti-inflammatory, immunomodulators, antiallergy, and antiviral agents [16, 17]**.**

Sea Urchins belong to Phylum Echinodermata, Subphylum Eleutherozea, Class Echinoidea. Sea urchins contain high levels of antioxidants such as polyphenols [18]. Dalian purple sea urchin (*Strongylo-centrotus nudus)* has been reported to have an anticancer and anti-fatigue effect [19, 20] as well as, antibiotic, antiviral, antiprotozoal and antifungal agents [21]. The gonads of some sea urchin species contain carotenoids such as astaxanthins, known to have neuroprotective activity. These carotenoids are produced by microalgae, while several marine invertebrates can bioaccumulate or synthesize it from metabolic precursors [22]. Sea Urchins could therefore be of great benefit in the pharmaceutical and food industries. Previous studies [23] showed that Sea Urchins extract contained a good number of phenolic compounds, such as Bisabolol oxide, Oleic acid, Hexadecanoic acid. Other compounds included Eugenol and Levomenthol. In vitro and in vivo studies revealed a high safety profile for the sea Urchin extract.

The composition varies considerably among different species of sea urchins due to their natural diet and physiological processes [24]. The biological activity of different species of sea urchins may also depend on the bioactive contents. Some species of sea urchins were found to have antimicrobial and antiviral activity (*Diadema antillarum, Tripneustes depressus,* and *Tripneustes ventricosus*) [25, 26], anti-inflammatory (*Evechinus chloroticus* and *Stomopneustes variolaris*) [27, 28], hepatoprotective (*Paracentrotus lividus*) [29], as well as anti-cardiotoxic (*Scaphechinus mirabilis*) [30].

To the best of our knowledge there are no studies reporting neuroprotective activity of sea urchins in a similar model of Cisplatin induced neurotoxicity. Therefore the present study investigates the use of sea Urchins crude extracts as a novel therapeutic agent to attenuate Cisplatin-induced neurotoxicity.

## Material and methods

### Animals

Male Sprague-Dawley (SD) rats (150–200 g body weight) were housed under standard conditions with water and food ad libitum. Rats were randomly divided into control and experimental groups (*n* = 6). Researchers were blinded to the experimental groups. All procedures were performed in compliance with the national institute of health (NIH) guidelines for the Care and Use of Laboratory Animals.

### Extracts preparation

Sea Urchins (*Diadema savignyi*) spine and shell extracts were prepared and characterized as previously described [23, 31]. Briefly, spines and shells were weighed (257, 304 g), and the fine powders were suspended in pure ethanol (1:1/w:v) and stirred continuously in the dark for 24 h using a magnetic stirrer. Both extracts were centrifuged at 8000 RPM for 15 minutes at 4 °C, and the resulting supernatants were concentrated by a rotary evaporator working under a vacuum at 50 °C. The extracts were stored at − 20 °C until the time of use.

### Experimental design

Rats were divided into six groups (*n* = 6 for each group). There were no sample size differences. Animals received either saline or Cisplatin (Mylan, France, 2 mg/kg body weight) intraperitoneally twice weekly for 4 weeks. Animals were treated concomitantly with either Urchins shell extract intraperitoneally (50 or 100 mg/kg, twice weekly) or Urchins spine extract (50 or 100 mg/kg) for 4 weeks.

Rats were divided into the following six groups:

- Group 1 (control): Injected intraperitoneally (i.p.) with saline.
- Group 2: received i.p. injection of Cisplatin (2 mg/kg body wt) twice a week for 4 weeks.
- Group 3 (SH 100): Injected with cisplatin as in group 2 in addition to Urchins’ shell extract at a dose of 100 mg/kg (high dose) concomitantly for 4 weeks.
- Group 4 (SH 50): Injected with cisplatin as in group 2 in addition to Urchins’ shell extract at a dose of 50 mg/kg (low dose) concomitantly for 4 weeks.
- Group 5 (SP 100): Injected with cisplatin as in group 2 in addition to Urchins’ spine extract at a dose of 100 mg/kg (high dose) concomitantly for 4 weeks.
- Group 6 (SP 50): Injected with cisplatin as in group 2 in addition to Urchins’ spine extract at a dose of 50 mg/kg (low dose) concomitantly for 4 weeks.

At the end of the experiment, Rats were anesthetized by Ketamine/Xylazine (ketamine 80–100 mg/kg, xylazine 10–12.5 mg/kg IP). Blood was collected by cardiac puncture, and tissue samples were collected for biochemical and histological analysis.

### Behavioral tests

#### Tail withdrawal test

Tail withdrawal test [32] was used to test the response to heat stimuli. Briefly, the rats were loosely restrained, and the tail placed over a heated surface maintained at a constant temperature (50–55 °C). The time to elicit a withdrawal of the tail was recorded.

#### Cold response test

Rats were placed in an enclosure over wet ice (Temp 4 °C). The time taken to evoke nociceptive behavior such as shaking, jumping, or paw licking was recorded as the response time [33]. A cut-off stimulation time of 40 seconds was used in case of no response to avoid skin damage or rat injury [34]. The mean of three trials for each test was used in the study.

### Biochemical parameters

At the end of the experiment, rats were anesthetized with Ketamine/Xylazine (ketamine 80–100 mg/kg, xylazine 10–12.5 mg/kg IP). Blood samples were collected and were allowed to clot. Serum was collected by centrifugation at 4000 rpm for 5 min. The collected serum was used to assess the concentrations of liver enzymes Alanine and Aspartate aminotransferases (ALT and AST), and the concentrations of serum creatinine and blood urea were measured by clinical chemistry autoanalyzer Mindray BS-240, China [35].

### Antioxidant and oxidative stress assays

Frozen brain tissue samples were homogenized in cold 0.1 M phosphate buffer using sonicator at 50 kHz for 2 mins per sample to separate homogenates by centrifugation at 4000 rpm for 10 min at 4 °C [36]. The supernatants were divided into aliquots to be used for measuring the oxidative stress and antioxidants markers including lipid peroxide (MDA), reduced glutathione (GSH), catalase (CAT) and nitric oxide (NO).

#### Malondialdehyde assay

The concentration of Malondialdehyde (MDA) as an indicator of membrane lipid peroxidation was analyzed using a colorimetric method (Biodiagnostics, Giza, Egypt) [37]. The assay is based on measuring thiobarbituric reactive substances result from the reaction of two molecules of thiobarbituric acid with one molecule of MDA in the acidic medium at a temperature of 95 °C for 25 min; the absorbance of sample (ASample) against blank and standard (AStandard) was measured at 535 nm.

#### Nitric oxide assay

The levels of Nitric oxide were measured in the brain tissue using Griess Method [38]. It is based on the use of nitrate reductase enzyme to convert nitrate to nitrite, which gives an azo-colored dye product that could be determined and measured at 540 nm.

#### Reduced glutathione assay

Reduced glutathione (GSH) level was estimated using a commercial assay kit (Biodiagnostic, Giza, Egypt [36]). The method based on the reduction of 5, 5 dithiobis-(2-nitrobenzoic acid) “Ellman’s reagent” (DTNB) with glutathione to produce a yellow compound. The reduced chromogen absorbance is directly proportional to GSH concentration. The absorbance was measured at 405 nm.

#### Catalase activity assay

Activity of catalase in brain tissues can be measured [39] by the addition of peroxidase (H2O2) which reacts with 4-aminophenazone (AAP) and 3,5-dichloro − 2-hydroxybenzene sulfonic acid (DHBS), forming a chromophore color that is inversely proportional to the amount of catalase. A commercial assay was used (Catalase Assay kit, Biodiagnostic, Giza, Egypt.).

### Histological studies

The brain was washed in PBS and fixed in 4% paraformaldehyde for 24 hours. Tissues were dehydrated in alcohol, cleared in terpineol for 24 hours, and infiltrated in Paraplast at 60 °C for 2 hours. Finally, the tissues were embedded in paraffin wax then sectioned using Leica Rotary Microtome TM (Model: 1512). Sections 5–6 μm thick were mounted on clean glass slides. Then paraffin sections were deparaffinized and stained with Mayer’s Hematoxylin and counterstained with 1% Eosin (H&E) [40]. Slides were examined using an Olympus light microscope (BX43, CellSence dimensions software).

The quantitative analysis of neurons in the sensorimotor cerebral cortex was performed by ImageJ® software as previously described [41]. Briefly, Neurons were randomly counted using 40x objective lens in 3 sections from every control and treated group. The density of neurons was calculated in 5 μm thick sections of the sensorimotor cerebral cortex in an area of 1334 μm^2^. The density in mm^3^ was calculated as neurons per field / (1334 X 1334 X (5 X10^− 9^)) = cells /mm^3^. The density of neurons in the control group was equal to 72/ (1334 * 1334 * 5 *10^− 9^ mm^3^) = 8091 cells/mm^3^ or 40.45 cells/mm^2^. The results were presented as the mean ± standard deviation.

### Immunohistochemical assay

Five microns thick paraffin-embedded tissue sections were prepared. Deparaffinized tissue sections were treated with 3% H2O2 for 20 Mins. Followed by incubation with anti Bcl2: PA5–11379 (Thermo Fisher Scientific Co.) Anti-Glial Fibrillary Acidic Protein (GFAP), by using a monoclonal antibody kit (Cat. No.13–0300-Thermo-scientific Co. Waltham, MA, USA) 1:100 overnight at 4C; washing followed by incubation with secondary antibody HRP Envision kit (DAKO) 20 mins; washing by PBS (phosphate buffer saline) and incubated with 3,3 diaminobenzidine (DAB) for 10 mins, then cover slipped for microscopic examination [42].

Six non-overlapping fields were randomly scanned per tissue section of each sample for the determination of the relative area percentage of immunohistochemical expression levels of Bcl2 and GFAP in the cerebral cortex of different groups. Morphological measurements and analyzed data were obtained using Leica Application module for tissue section analysis attached to a Full HD microscopic imaging system (Leica Microsystems GmbH, Germany) [42].

### Statistical analysis

All data were analyzed using version 7.00 GraphPad Prism Software Inc. (San Diego, CA, USA). Data were expressed as the mean ± standard deviation (SD), and *P*-values < 0.05 were considered statistically significant. One-way ANOVA test was followed by Tukey test for multiple comparisons.

## Results

### Behavioral assessment

#### Heat sensitivity

**Fig. 1:**
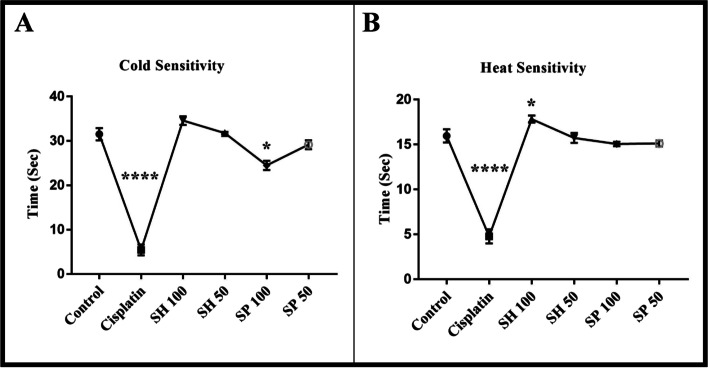
Behavioral Assessment. Cisplatin administration significantly decreased cold withdrawal latency for cold (**a**) from about 30 sec to about 5 seconds (**** *p*-value < 0.0001). Treatment with shell and spine extracts increased the latency back to control levels, except for the SP-100 group which was still below the control levels. (*p-value < 0.05, *n* = 6). Heat withdrawal latency decreased from 15 sec to about 5 seconds (**** *p*-value < 0.0001) following Cisplatin administration. Treatment with shell and spine extracts increased the latency back to control levels, except for the SH-100 group which was longer than control levels. (*p-value < 0.05, *n* = 6)

The administration of cisplatin resulted in significant behavioral changes. A decrease in withdrawal latencies in heat tests from 15 seconds to 5 seconds (Fig. 1a) was observed after cisplatin administration (****p-value < 0.0001) indicating the development of hyperalgesia. Treatment with both urchin’s extracts caused a significant improvement in withdrawal time, with the best effect seen in the shell SH-100 group (*p-value < 0.05).

#### Cold sensitivity

The cold sensitivity test showed a dramatic decrease in the latency from approx. 30 seconds to about 5 seconds after treatment with cisplatin (****p-value < 0.0001). The administration of both Urchins’ extracts SH 50–100 and SP 50 (Fig. 1b) reversed the cisplatin effect and brought the withdrawal latencies back to normal levels (29.15–34.58). Although the spine SP-100 dose showed a slightly lower effect (*p-value < 0.05).

### Biochemical parameters

#### Liver and kidney function enzymes

**Fig. 2:**
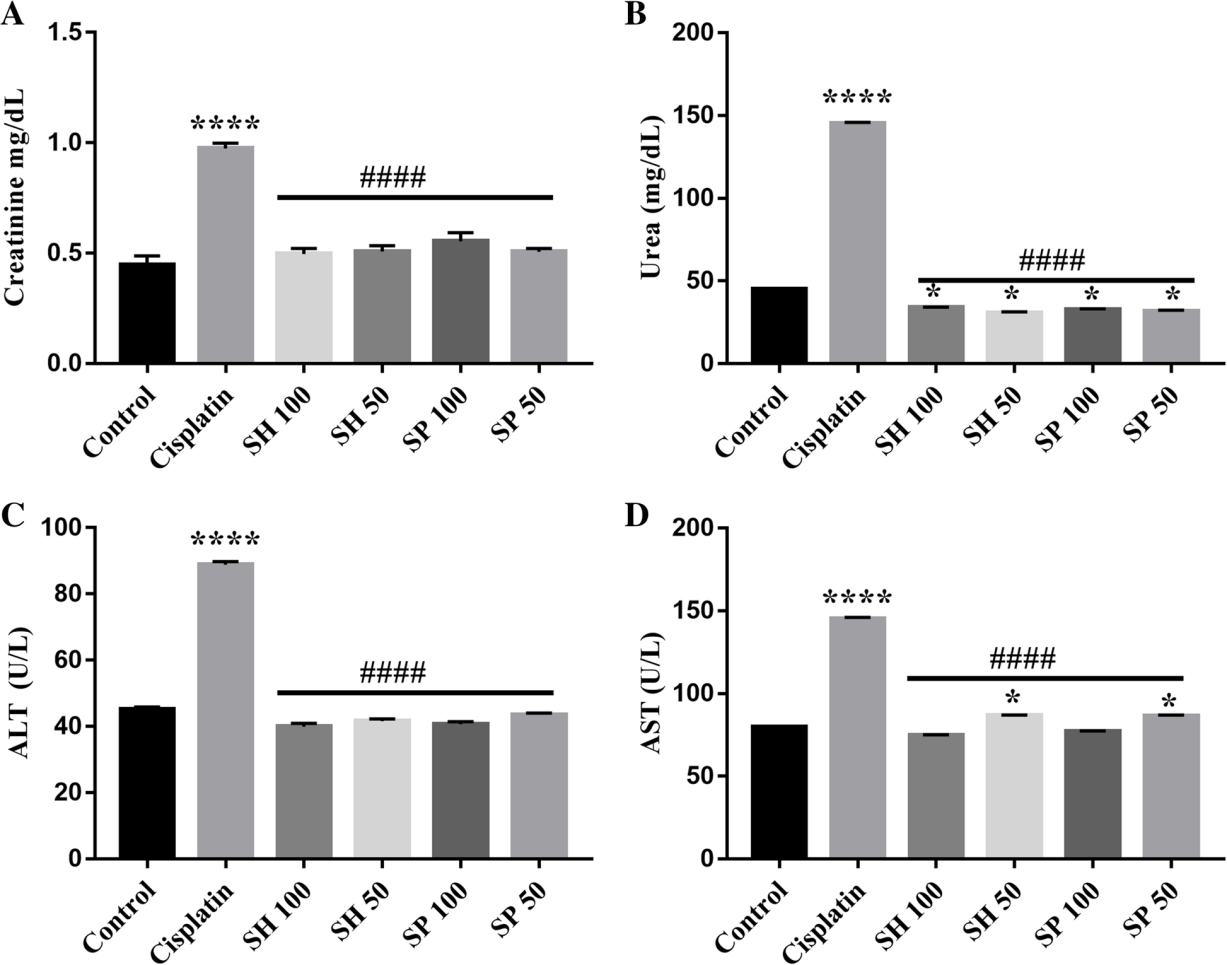
Kidney and liver function tests. Cisplatin administration increased ALT, AST, Urea and Creatinine (**** *p*-value < 0.0001). Treatment with sea Urchin extracts reduced Creatinine levels below those of the cisplatin treated group (#### *p*-value < 0.0001). There was no significant difference between the different treated groups and control. On the other hand, Urea levels were significantly lower than normal control and cisplatin group (*p-value < 0.05, #### *p*-value < 0.0001). ALT and AST were also reduced back to normal levels. The AST levels in the SH and SP 50 mg treatment groups were significantly higher than normal (*n* = 5, *p-value < 0.05)

Kidney function tests showed that cisplatin caused an increase by 117.24% in creatinine and urea levels (**** *p*-value < 0.0001, Fig. 2a & b). No significant changes in creatinine levels were seen in the different urchin’s shell (SH) and spine (SP) treated compared to the control group (Table 1). On the other hand, treatment with shell and spine extracts showed a significant difference in Urea levels (*p-value < 0.05) between the treated groups and the control.

**Table 1:** Kidney and liver function tests

	Creatinine	Urea	ALT	AST
**Control**	0.4483 ± 0.039^**b**^	44.94 ± 0.065^**b**^	45.22 ± 0.685^**b**^	79.8 ± 0.2^**b**^
	SEM ± 0.022	SEM ± 0.037	SEM ± 0.306	SEM ± 0.089
**Cisplatin**	0.974 ± 0.024^**a**^	145.5 ± 0.51^**a**^	88.91 ± 0.91^**a**^	145.8 ± 0.55^**a**^
	SEM ± 0.014	SEM ± 0.28	SEM ± 0.407	SEM ± 0.246
**SH 100**	0.4967 ± 0.025^**b**^	33.88 ± 0.12^**a,b**^	39.99 ± 0.99^**b**^	74.5 ± 0.5^**b**^
	SEM ± 0.013	SEM ± 0.069	SEM ± 0.44	SEM ± 0.226
**SH 50**	0.5077 ± 0.026^**b**^	30.65 ± 0.65^**a,b**^	41.65 ± 0.65^**b**^	86.8 ± 0.2^**a,b**^
	SEM ± 0.015	SEM ± 0.37	SEM ± 0.290	SEM ± 0.083
**SP 100**	0.5543 ± 0.038^**b**^	32.7 ± 0.69^**a,b**^	40.73 ± 0.725^**b**^	76.87 ± 0.41^**b**^
	SEM ± 0.022	SEM ± 0.40	SEM ± 0.324	SEM ± 0.21
**SP 50**	0.5073 ± 0.015^**b**^	31.78 ± 0.71^**a,b**^	43.5 ± 0.51^**b**^	86.4 ± 0.56^**a,b**^
	SEM ± 0.018	SEM ± 0.44	SEM ± 0.223	SEM ± 0.25

Data is expressed as mean ± SD, and Standard error of the mean SEM

^a^Significant compared to the control group; *p*-value < 0.0001. ^b^Significant compared to the Cisplatin model group; *p*-value < 0.0001

Liver enzymes revealed that cisplatin caused a dramatic increase in ALT by 96.63% and AST levels by 82.64% (**** *p*-value < 0.0001, Fig. 2c & d). Treatment with urchin extracts significantly decreased ALT and AST levels back to normal. There were no significant differences between the sea Urchin’s treated groups (SH and SP) compared to the control group, except for AST in SH 50 (8.77%) and SP 50 (8.45%) treated groups (*p-value < 0.05, Fig. 2d).

#### Hematological parameters

**Fig. 3:**
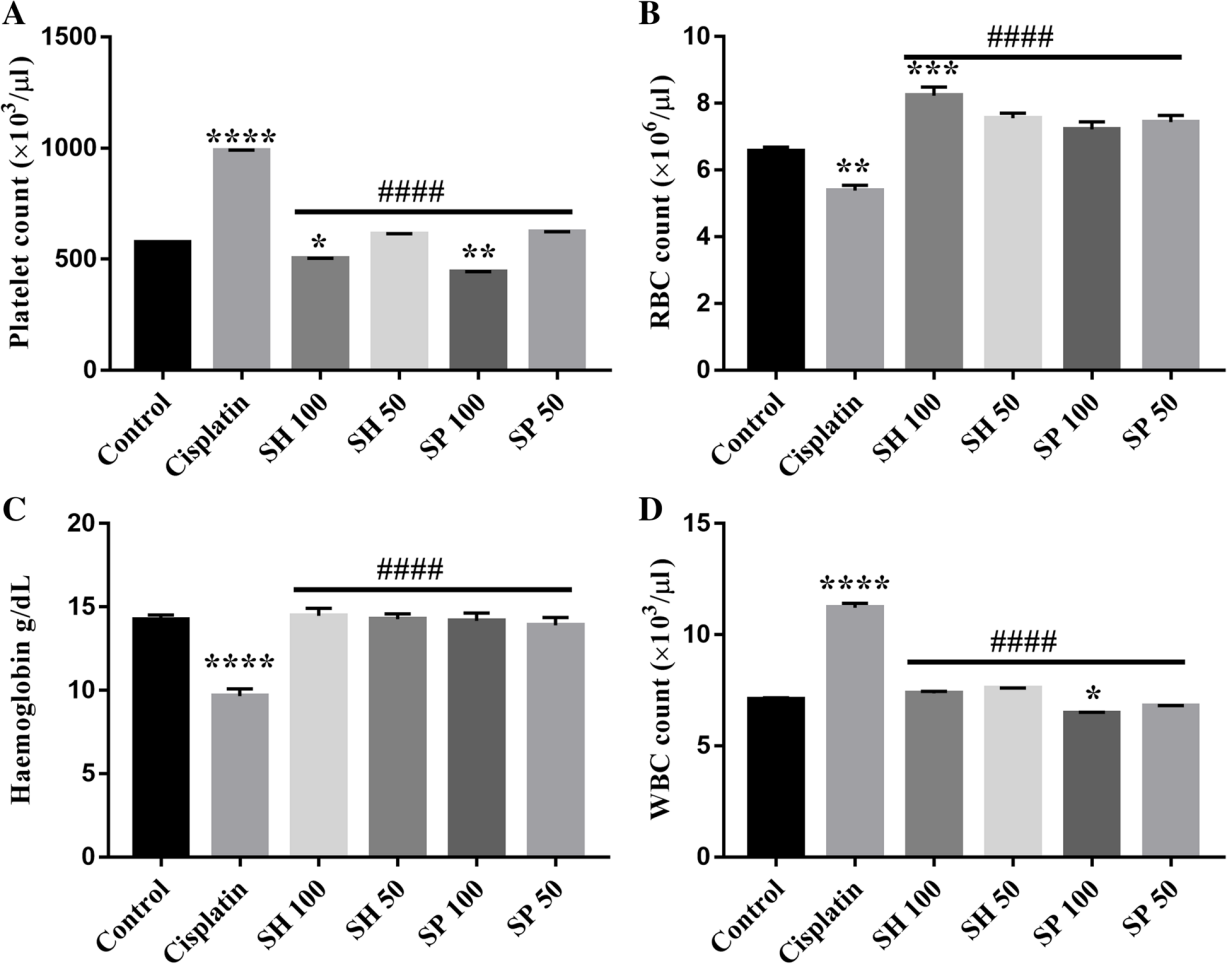
Hematological parameters. Cisplatin administration caused significant changes in hematological parameters Hemoglobin, red blood cell (RBC), white blood cells (WBC), and platelets. Treatment with both spine and Shell extracts reversed these changes. The shell 100 mg treatment group showed higher levels of RBCs compared to normal. In contrast, the spine 100 mg treatment caused a reduction in WBCs below normal levels. *n* = 6, *p-value < 0.05; **p-value < 0.01; ***p-value < 0.001; **** *p*-value < 0.0001. Platelets, RBCs, WBCs, and Hemoglobin levels were significantly different from those of the cisplatin treated group (#### *p*-value < 0.0001)

Cisplatin administration caused a significant reduction in Hemoglobin levels and red blood cell (RBC) count compared to control (30.92 and 19.45% respectively, ****p-value < 0.0001, **p-value < 0.01, Fig. 3a & b and Table 2). Treatment with both urchin’s extracts (SH 50–100 and SP 50–100) reversed the cisplatin effect. Specifically SH-100 treated group showed a significant increase in the RBC’s count (***p-value < 0.001).

**Table 2:** Hematological parameters

	Hemoglobin	RBC	WBC	PLT
**Control**	14.24 ± 0.28 ^**b**^	6.56 ± 0.12^**b**^	7.10 ± 0.072^**b**^	576 ± 1.84^**b**^
	SEM ± 0.16	SEM ± 0.07	SEM ± 0.036	SEM ± 0.82
**Cisplatin**	9.65 ± 0.43^**a**^	5.38 ± 0.15^**a**^	11.22 ± 0.20^**a**^	989.7 ± 1.56^**a**^
	SEM ± 0.255	SEM ± 0.09	SEM ± 0.10	SEM ± 0.77
**SH 100**	14.48 ± 0.44^**b**^	8.22 ± 0.26^**a,b**^	7.36 ± 0.096^**b**^	502.9 ± 1.63^**a,b**^
	SEM ± 0.25	SEM ± 0.14	SEM ± 0.048	SEM ± 0.81
**SH 50**	14.27 ± 0.32^**b**^	7.55 ± 0.15^**b**^	7.6 ± 0.008^**b**^	613.5 ± 1.76^**b**^
	SEM ± 0.018	SEM ± 0.08	SEM ± 0.004	SEM ± 0.88
**SP 100**	14.10 ± 0.45^**b**^	7.22 ± 0.22^**b**^	6.467 ± 0.04^**a,b**^	443 ± 1.19^**a,b**^
	SEM ± 0.026	SEM ± 0.13	SEM ± 0.023	SEM ± 0.59
**SP 50**	13.91 ± 0.46^**b**^	7.43 ± 0.20^**b**^	6.8 ± 0.012^**b**^	622.9 ± 1.25^**b**^
	SEM ± 0.27	SEM ± 0.12	SEM ± 0.006	SEM ± 0.62

Data is expressed as mean ± SD, and Standard error of the mean SEM

^a^Significant compared to the control group; *p*-value < 0.0001. ^b^Significant compared to the Cisplatin model group; *p*-value < 0.0001

White blood cells (WBC) and platelets count showed a significant increase in cisplatin group compared to the control (60.13 and 71.10% respectively, ****p-value < 0.0001, Fig. 3c & d). Both urchin’s extracts treated groups did show significant improvement of the WBC count except for SP-100 which was significantly decreased from control levels (8.52%, *p-value < 0.05). Urchin’s extracts SH-100 and SP-100 significantly reduced platelets count (12.8 and 23.18%, *p-value < 0.05, **p-value < 0.01) compared to the Cisplatin group.

#### Oxidative stress markers

**Fig. 4:**
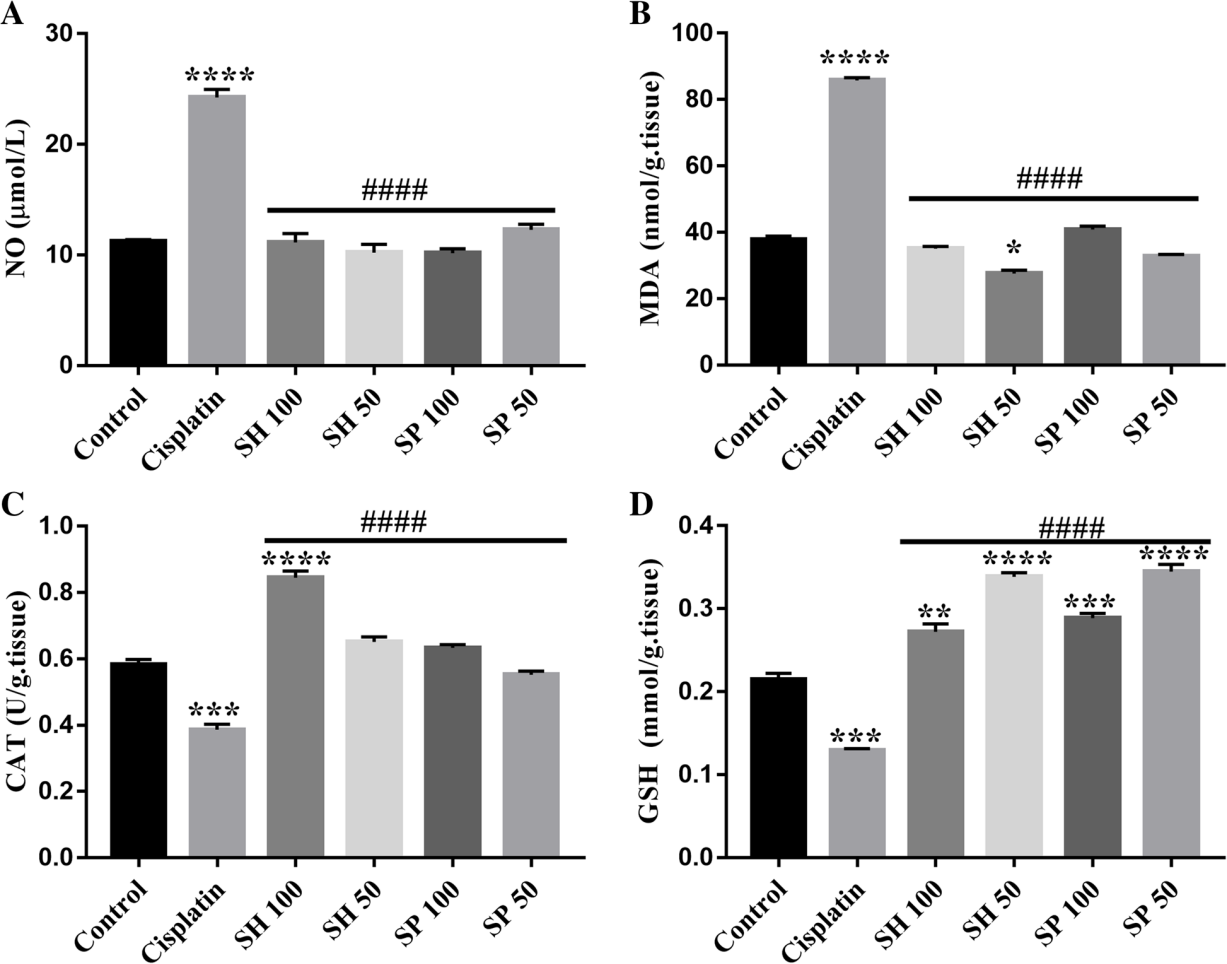
Oxidative Stress Markers. Cisplatin administration caused a significant increase in Nitrous oxide (NO) levels and Malondialdehyde (MDA) levels in the brain tissue. Treatment with both extracts caused a reduction of these levels back to normal. Reduced glutathione (GSH) and Catalase activity (CAT) levels were significantly lower than normal in the Cisplatin group. Treatment with Urchin extracts increased the levels of GSH significantly above normal levels. NO, MDA, CAT, and GSH levels were significantly different from those of the cisplatin treated group (#### *p*-value < 0.0001). (*n* = 6, *p-value < 0.05; **p-value < 0.01; ***p-value < 0.001; **** *p*-value < 0.0001)

The levels of nitric oxide (NO) and malondialdehyde (MDA) increased significantly in the cisplatin-treated rats compared to the control group (116.37 and 126.68% respectively, ****p-value < 0.0001, *p-value < 0.05, Fig. 4a & b and Table 3).

**Table 3:** Oxidative stress markers

	NO	MDA	GSH	CAT
**Control**	11.21 ± 0.135^**b**^	37.85 ± 0.74^**b**^	0.215 ± 0.005^**b**^	0.582 ± 0.01^**b**^
	SEM ± 0.077	SEM ± 0.42	SEM ± 0.002	SEM ± 0.006
**Cisplatin**	24.25 ± 0.51^**a**^	85.8 ± 0.59^**a**^	0.129 ± 0.001^**a**^	0.386 ± 0.01^**a**^
	SEM ± 0.29	SEM ± 0.34	SEM ± 0.0008	SEM ± 0.006
**SH 100**	11.13 ± 0.57^**b**^	35.17 ± 0.43^**b**^	0.272 ± 0.007^**a,b**^	0.845 ± 0.014^**a,b**^
	SEM ± 0.32	SEM ± 0.25	SEM ± 0.004	SEM ± 0.008
**SH 50**	10.22 ± 0.53^**b**^	27.62 ± 0.72^**a,b**^	0.33 ± 0.003^**a,b**^	0.651 ± 0.01^**b**^
	SEM ± 0.3	SEM ± 0.41	SEM ± 0.002	SEM ± 0.006
**SP 100**	10.16 ± 0.28^**b**^	40.92 ± 0.69^**b**^	0.289 ± 0.004^**a,b**^	0.632 ± 0.007^**b**^
	SEM ± 0.17	SEM ± 0.40	SEM ± 0.002	SEM ± 0.004
**SP 50**	12.3 ± 0.355^**b**^	32.86 ± 0.36^**b**^	0.345 ± 0.006^**a,b**^	0.552 ± 0.008^**b**^
	SEM ± 0.20	SEM ± 0.21	SEM ± 0.003	SEM ± 0.004

Data is expressed as mean ± SD, and Standard error of the mean SEM

^a^Significantly different from the control group; *p*-value < 0.0001. ^b^Significantly different from the Cisplatin model group; *p*-value < 0.0001

Treatment with both urchin’s extracts reversed the changes in NO and MDA back to the normal control levels. Except for the SH-50 group where MDA was significantly lower than control (*p-value < 0.05, Fig. 4b).

Reduced glutathione levels (GSH) and catalase activity (CAT) showed a significant decrease in cisplatin-treated rats compared to the control group (39.76 and 33.59% respectively, ***p-value < 0.001, Fig. 4c & d and Table 3). There was no significant change in catalase activity between the control, spine (SP 50–100), and shell (SH 50) groups. The shell 100 mg group on the other hand showed a significant increase in CAT activity (45.1%, ****p-value < 0.0001).

Reduced GSH activity was significantly elevated in all treated groups, (SH 100, 26.5%, **p-value < 0.01, and SP 100, 34.4%, ***p-value < 0.001),. The SH 50, (and SP 50, 57.44 and 60.46% respectively were also significantly higher than cisplatin and control groups ****p-value < 0.0001.

#### Histological investigations

**Fig. 5:**
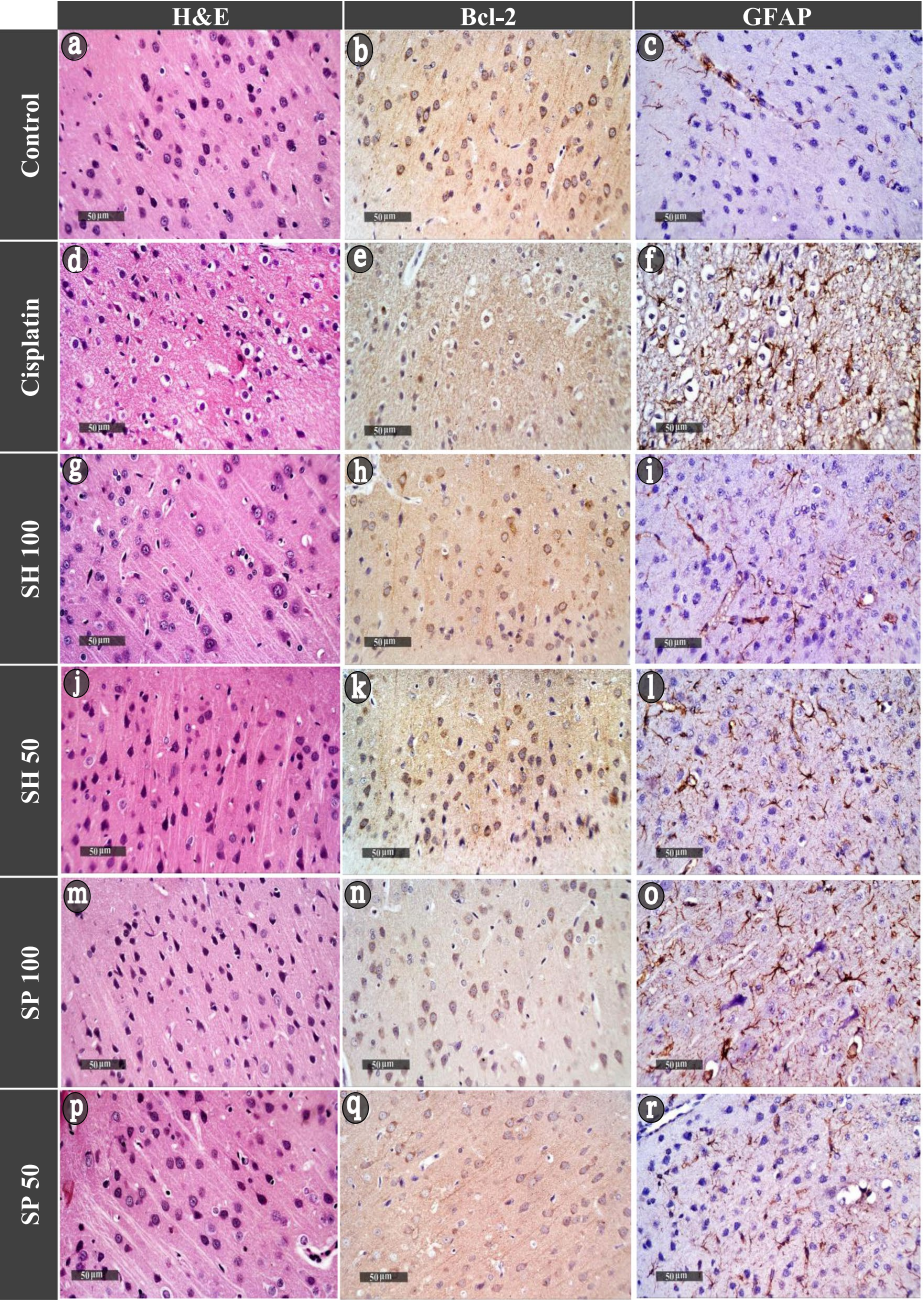
Histological evaluation. Photomicrographs of brain sections showing the cerebral cortex of control rats (**a, b, c**) stained with hematoxylin and eosin (**a**) illustrating neuronal populations within the cortex are normally heterogeneous mixtures of many granular cells. Immuno-stained sections showed normal positive expression of Bcl2 in almost all apparent neurons (**b**), remarkably without apoptotic cells. Normal levels of expression of GFAP (**c**) immunostaining. Cisplatin-treated rats (**d, e, f**) showed neuronal degenerative changes particularly karyopyknosis and shrunken necrotic ghost like neurons. Bcl2 (**e**) immuno-stained sections showed negative Bcl2 indicating apoptotic cells. A strong positive GFAP (**f**) reaction and hypertrophic astrocytes indicating severe astrogliosis. Shell extract treated groups (100 mg/kg, **g, h, i**, and 50 mg/kg, **j, k, l**) showed intact homogenous neuropil with normal granular neurons and minimal reactive neuroglial cells infiltrates. Immuno-stained sections showed few apoptotic cells (high Bcl2 positive reaction), and mild astrogliosis. Spine extracts group 100 mg/kg (**m, n, o**) showed mild vacuolization of neuropil with pyknotic satellite cells and loss of normal pyramidal or fusiform shape of the neuron. Immuno-stained sections showed low positive reaction in most apparent neurons, more apoptotic cells and reactive astrogliosis evidenced. The 50 mg/kg spine treated group (**p, q, r**) showed normal pyramidal cells. Immuno-stained sections showed few apoptotic (Bcl2 positive) cells with normally distributed GFAP stained astrocytes

Examination of the brain sections stained with hematoxylin and eosin of the control group revealed normal cerebral cortex architecture, with a mixture of many granular cells with basophilic cytoplasm and central vesicular nuclei. Cisplatin treatment caused neuronal degenerative changes manifested by karyopyknosis and shrunken necrotic ghost like neurons, as well as vacuolization of neuropil with moderate higher reactive neuroglial cell infiltrates. Treatment with Urchin shell extracts showed neuroprotection as evidenced by the intact homogenous neuropil with normal granular neurons, shrunken hyperchromatic basophilic perikaryon also and minimal reactive neuroglial cells infiltrates. In the spine extract treated group (SP-100) mild vacuolization of neuropil was observed with remarkable pyknotic satellite cells and loss of normal pyramidal or fusiform shape of the neuron with co-persistence of reactive neuroglial cells infiltrates (Fig. 5).

**Fig. 6:**
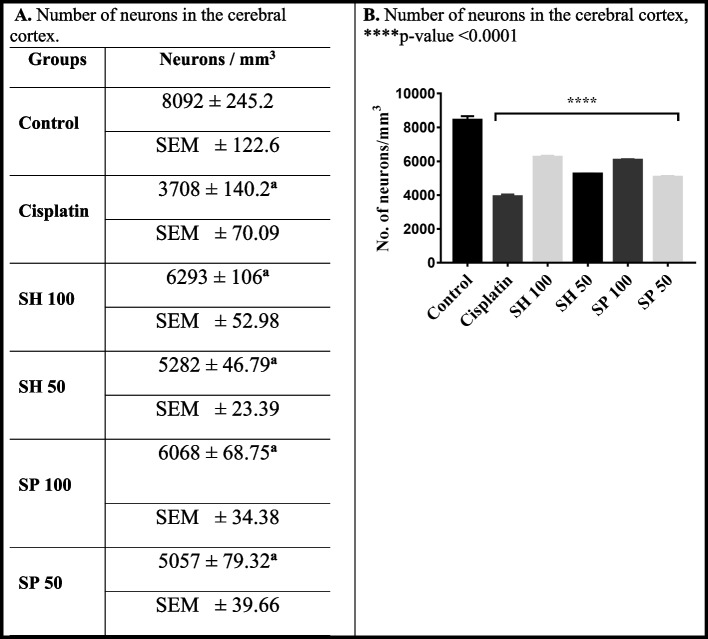
Counting of the cerebral cortex neurons showed that Control rats showed an average of 8091 cells / mm^3^ compared to the Cisplatin group, which showed 3708 cells/ mm^3^. Sea Urchins Shell extract treatment showed an average of 5282 and 6293 cells / mm^3^ in the 50 mg and 100 mg groups respectively. Spine treated groups were showed higher neuron counts at 5057 and 6068 cells / mm^3^ in the 50 mg and 100 mg groups, respectively (*n* = 6, *p*-value < 0.0001). **a** Significantly different from the control group; *p*-value < 0.0001. **b** Significantly different from the Cisplatin model group; *p*-value < 0.0001. Data is expressed as mean ± SD, and Standard error of the mean SEM

Quantification of neurons in the sensorimotor cerebral cortex (Fig. 6) showed an average of 8091 cells / mm^3^ in normal control compared to the Cisplatin group, which showed 3708 cells/ mm^3^. Shell extract treatment showed an average of 5282 and 6293 cells / mm^3^ in the 50 mg and 100 mg groups respectively, while the Spine treated groups showed slightly higher neuronal numbers (5057 and 6068 cells / mm^3^ in the 50 mg and 100 mg groups, respectively, *p*-value < 0.0001).

#### Immunohistochemistry

Immuno-histochemical examination of the control rats cerebral cortex showed normally distributed astrocytes stained with Glial fibrillary acidic protein (GFAP) without pronounced overlap of astrocyte processes. Tissue sections stained with the apoptotic marker B-cell lymphoma 2 (Bcl2), showed a high positive reaction in almost all apparent neurons without any remarkable apoptotic cells (Fig. 5b, c).

Cisplatin-treated group: showed strong positive GFAP reaction and hypertrophic astrocytes with multiple elongated processes indicating severe astrogliosis. This was in the form of astrocytic proliferation associated with increased size, number, and length of their cell body as well pronounced overlap of their processes**.** Examination of Bcl2 sections showed a negative reaction in most apparent neurons, almost the whole field were apoptotic cells surrounded by a halo with high reactive disrupted glial cells (Fig. 5e, f).

Examination of brain sections in SH-100 and SH-50 groups showed mild astrogliosis manifested by the preservation of individual astrocytes without pronounced overlap of astrocyte processes. While the investigation of antiapoptotic activities of the neurons revealed high Bcl2 positive reaction in most apparent neurons and few glial cells as well as few apoptotic cells (Fig. 5h, I, and k, l).

Examination of brain sections from SP-100 group displayed diffuse reactive astrogliosis evidenced by the pronounced overlap of astrocyte processes with moderate expression for GFAP. Investigation of Bcl2 sections showed low positive reaction in most neurons, and more apoptotic cells with high reactive disrupted glial cells (Fig. 5n, o). The SP-50 treated group revealed normally distributed astrocytes in-between, manifested by preservation of individual astrocyte and without pronounced overlap of astrocyte processes. Bcl2 staining showed high positive reaction in most apparent neurons, few apoptotic cells with little disrupted glial cells (Fig. 5q, r).

#### Quantification of immunohistochemistry

**Fig. 7:**
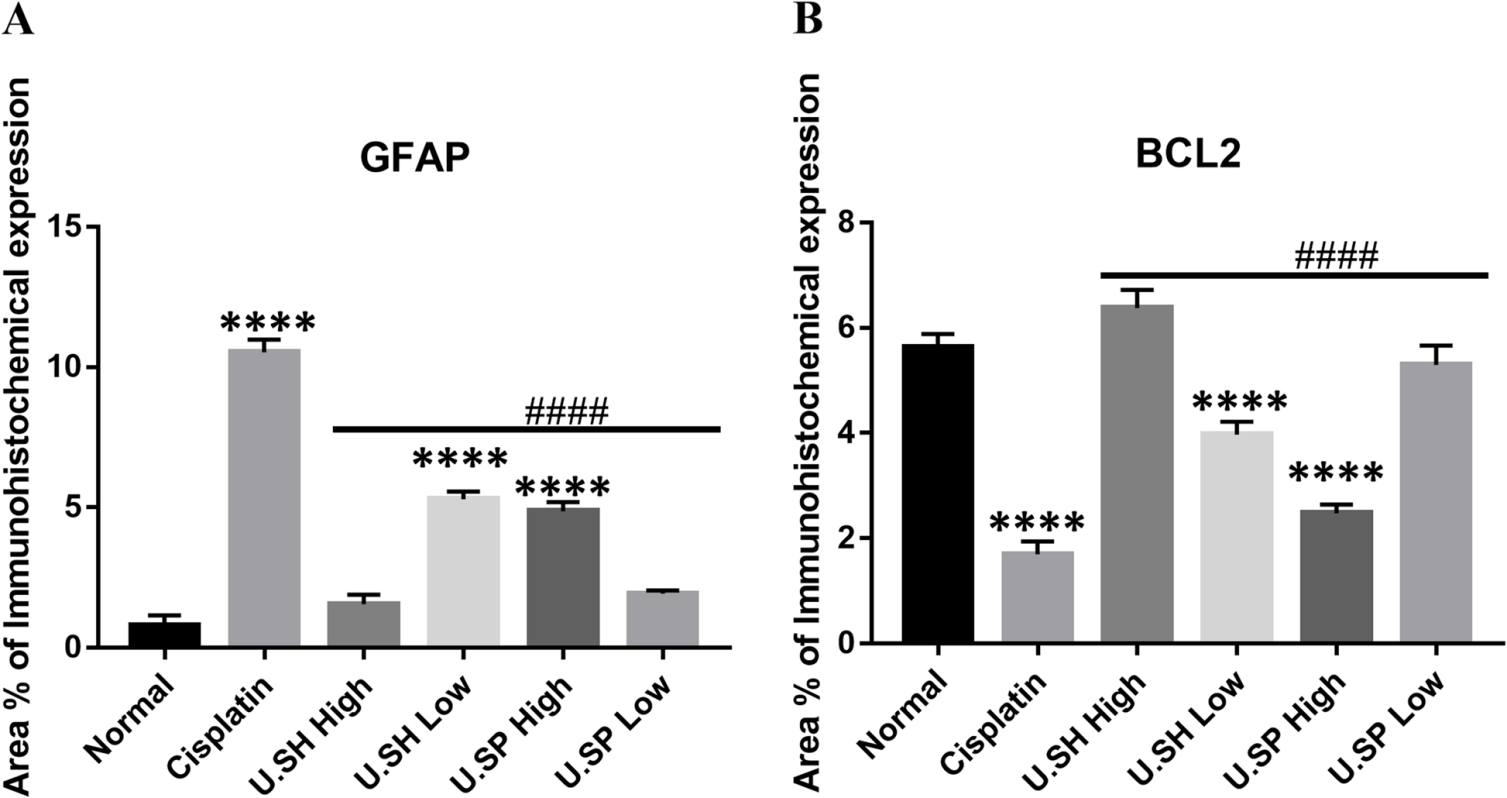
Immunohistochemical quantitative analysis showed the area of immuno-histochemical expression of GFAP (**a**) increased significantly after cisplatin administrations while the expression level of Bcl2 (**b**) decreased. Treatment with Spine (50–100) and Shell (50–100) extracts reversed these changes. Data presented as mean ± SD of the area (**** *p* < 0.0001). GFAP, and Bcl2 levels were significantly different from those of the cisplatin treated group (#### *p*-value < 0.0001)

Quantitative analysis of the immuno-histochemical expression of GFAP showed a 10-fold increase after cisplatin administration. Treatment with SH 100 and SP 50 caused a dramatic decrease of mean expression values. On the other hand, treatment with SH 50 and SP 100 caused a remarkable decline compared to the cisplatin group but the expression was still significantly higher than the control group (****P-value < 0.0001, Fig. 7a).

A decrease of anti-apoptotic marker Bcl2 in the cisplatin treated animals (****P-value < 0.0001) was seen. While the animals treated with SH 100 and SP 50 showed high immune expressions levels. (Fig. 7b).

## Discussion

Platinum-based drugs (Platins) such as Cisplatin, oxaliplatin, and Carboplatin are commonly used in chemotherapy, which unfortunately, cause severe toxicity, which limits their usage. Platins induce oxidative damage, inflammation, mitochondrial dysfunction, DNA damage, and apoptosis [1, 10–12]. Toxicity of the nervous system causes pain and mechanical, thermal allodynia, and hyperalgesia [43].

Animal models have been used to assess neurotoxicity using behavioral, electrophysiologic, and histological methods. Behavioral tests used to assess mechanical, and thermal hyperalgesia [44], have shown sensory disorders after repeated injections of Cisplatin in rats [45]. These sensory changes are very similar to that observed in rats following treatment with other chemotherapeutic agents and following direct nerve injury [46, 47]. In the current study, rats treated with Urchins extracts showed improved cold and heat sensitivity, which is in line with previous reports [48] showing the anti-nociceptive activity of *Agrimonia pilosa* extract on the acetic acid-induced writhing, tail-flick, and hot-plate tests [48].

In the current study, treatment with Urchins extracts significantly improved the hematological parameters as well as the liver and renal functions, which proves the systemic effects of these extracts in reversing the cisplatin induced toxicity on other body systems [49–51].

It is suggested that Cisplatin causes toxic effects in humans and animals via lipid peroxidation and oxidative stress [52–57]. Oxidative stress often results from the disturbances in antioxidant defense enzymes such as superoxide dismutase (SOD), glutathione peroxidase and glutathione reductase, and catalase (CAT). The current study shows that Cisplatin produced a significant increase in the MDA levels, which confirms that it induces lipid peroxidation in the brain. Catalase enzyme activity and GSH levels were also reduced after Cisplatin treatment [54]. The findings in this study show that Urchin’s treatment may possibly affect the oxidative process in the cells thus reversing the tissue damage in the liver, kidney, and the brain.

The gonads of sea Urchins contain carotenoids such as astaxanthins, known to have neuroprotective activity [22]. The shell and spine of the purple sea urchin *Echinometra mathaei* obtained from the Persian Gulf also contain carotenoids such as astaxanthins, known to have neuroprotective activity [31, 58, 59]. The biological effects of the urchins extracts are due to the bioactive peptides in the extracts, such as terpenoids, phenolic acids, and naphthoquinones. Oxidative stress often leads to inflammation and tissue damage thus disturbing the homeostasis of different organ systems in the body. Cyclooxygenases (COX) and lipoxygenases (LOP) are two enzymes families that play a major role in the inflammatory process [60]. Reports indicate that 4-hydroxyl- 1-(16-methoxiprop-16-en-15-yl)-8-methyl-21, 22-dioxatricyclo [11.3.1.15,8] octadecane- 3,19-dione, a diterpenoid isolated from the gonads of S. variolaris, inhibits COX-2 and 5-lipoxygenase (LOP-5) enzymes thus disrupting and potentially reversing the inflammatory process and the resulting tissue damage. The extracts of the gonads of some sea urchin species could therefore play an essential role as anti-inflammatory and antioxidants in many conditions in which the pathology is attributed to these mechanisms, such as Diabetes,

Histological assessment of the brain tissue showed that Cisplatin causes neuronal damage in the cerebral cortex and cellular infiltration [6, 61, 62]. Consistent with previous reports these abnormalities were significantly alleviated by the treatment of both sea Urchin extracts [63, 64].

Immunostaining using Bcl2 and GFAP showed apoptotic cells and astrogliosis. Both extracts showed low apoptotic cell numbers and lower astrogliosis. Previous reports showed that exposure to platinum compounds markedly reduced Bcl-2 protein levels [65] possibly due to proteolytic cleavage by caspase3/7 or transcriptional downregulation, and enhanced Bax causing a decreased Bcl-2/Bax ratio, which is key regulator of apoptosis [66]. The hippocampal GFAP expression also increased significantly in a Streptozotocin induced model of Alzheimer’s disease, indicating a correlation between neuronal damage and glial cell infiltration [42, 67].

## Conclusion

The current study highlights the protective effect of Sea Urchins (*Diadema savignyi)* extracts in reversing the damage caused by cisplatin*.* Neuronal cell death and oxidative damage following Cisplatin administration were reversed after the administration of sea Urchins extracts. The extracts also reversed damage to the liver and kidney and reversed oxidative damage in the brain tissue. Further studies are recommended to identify the bioactive compounds of the extract and their molecular targets.

## Data Availability

All data generated or analysed during this study are included in this published article.
